# Association between the APEX1 Asp148Glu polymorphism and prostate cancer, especially among Asians: a new evidence-based analysis

**DOI:** 10.18632/oncotarget.9693

**Published:** 2016-05-29

**Authors:** Yang Chen, Jie Li, Zengnan Mo

**Affiliations:** ^1^ Center for Genomic and Personalized Medicine, Guangxi Medical University, Nanning, Guangxi Zhuang Autonomous Region, China; ^2^ Department of Urology and Nephrology, The First Affiliated Hospital of Guangxi Medical University, Nanning, China; ^3^ Research Center for Guangxi Reproductive Medicine, First Affiliated Hospital of Guangxi Medical University, Guangxi Zhuang Autonomous Region, China; ^4^ Guangxi key laboratory for genomic and personalized medicine, Guangxi collaborative innovation center for genomic and personalized medicine, Nanning, Guangxi Zhuang Autonomous Region, China

**Keywords:** APEX1, rs1130409, polymorphism, prostate cancer

## Abstract

**Background:**

Prostate cancer (Pca) is a serious disease associated with considerable morbidity and mortality. As a causative factor, the Asp148Glu polymorphism has been identified in the apurinic/apyrimidinic endonuclease (APEX1) gene. However, the association among Asians is considered controversial.

**Methods:**

Evidence for this association was obtained from the PubMed, Embase, HuGENet and Chinese National Knowledge Infrastructure (CNKI) databases. In the analysis, four models were applied. Associations between the APEX1 polymorphism and the invasiveness of Pca based on the Gleason score, prostate-specific antigen expression and clinical status were also evaluated.

**Results:**

Seven articles were included in the analysis. Positive results were not only discovered in the pooled analysis, but also among patients of mixed descentand Asian descent. However, after considering the Hardy-Weinberg equilibrium (HWE), we observed only a 1.557-fold increase in Pca risk for subjects of Asian descent(GG vs. TT: OR=1.557, 95%CI=1.069-2.268) under the co-dominant model. Additionally, we did not also find any relationship between the APEX1 Asp148Glu polymorphism and invasive Pca risk.

**Conclusion:**

On the basis of the function of the APEX1 Asp148Glu polymorphism, recent studies, and our results, we suggest that the APEX1 Asp148Glu polymorphism might be important in stimulating the development of Pca rather than its invasiveness in various populations, especially for Asians.

## INTRODUCTION

Prostate cancer (Pca) was one of the mostly frequently diagnosed malignant diseases worldwide. It is associated with considerable morbidity and is the second leading cause of cancer mortality in western countries [1–3]. In 2016, an estimated 180,890 new cases of Pca and 26,120 deaths are projected to occur in the United States [4]. Moreover, the incidence of Pca in China has been increasing [5]. As a multi-factorial disease, many factors are known to play a key role in Pca development, such as age, ethnicity, diet, and geographic factors [6]. Recently, hereditary factors have also been identified to be significantly associated with Pca [7], particularly regarding polymorphisms in the apurinic/apyrimidinic endonuclease (APEX1) gene [8].

The APEX1 gene is located on chromosome 14 from 20455131bp to 20457772bp. It encodes for an enzyme belonging to the base excision repair (BER) pathway, which is important in the repair of DNA damage caused by oxidative reagents and alkylation [9–10]. As one of the key genes in the BER pathway, APEX1 identifies and splits phosphodiester bonds *via* a hydrolytic mechanism on the 5′-side of abasic sites, thus specifically activating DNA repair [11]. This gene also participates in other crucial cellular processes, such as the response to oxidative stress, cell cycle control, and apoptosis [12]. In 2013, Pan et al. [13] suggested that the APEX1 gene might be one of risk factors contributing to the morbidity of lung cancer. In addition, other diseases, such as cervical cancer [14], ovarian cancer [15], and colorectal cancer [16] have also been associated with APEX1 gene polymorphisms.

There are several polymorphisms in the APEX1 gene, of which Asp148Glu (rs1130409) has been associated with many cancers [14–16, 23–25, 27]. In 2001, Hu et al. [33] conducted a study on the association between variants of APE1 and ionizing radiation, which suggested that the G allele transformed from T (Asp > Glu) is associated with mitotic delay in lymphocytes, providing greater sensitivity to ionizing radiation. Moreover, the G of allele rs1130409 was also found to increase the risk for the development of Pca [17, 22]. On the basis of the function of the APEX1 gene, it has been speculated that the G allele of the APEX1 Asp148Glu polymorphism might have an effect on normal DNA repair, and may play a role in inducing Pca. However, this association was considered controversial in follow-up studies [8], especially among Asians. In order to evaluate the real association, the latest and most convincing evidence was used in this meta-analysis.

## RESULTS

### Characteristics of the retrieval and eligible studies

**Figure 1:**
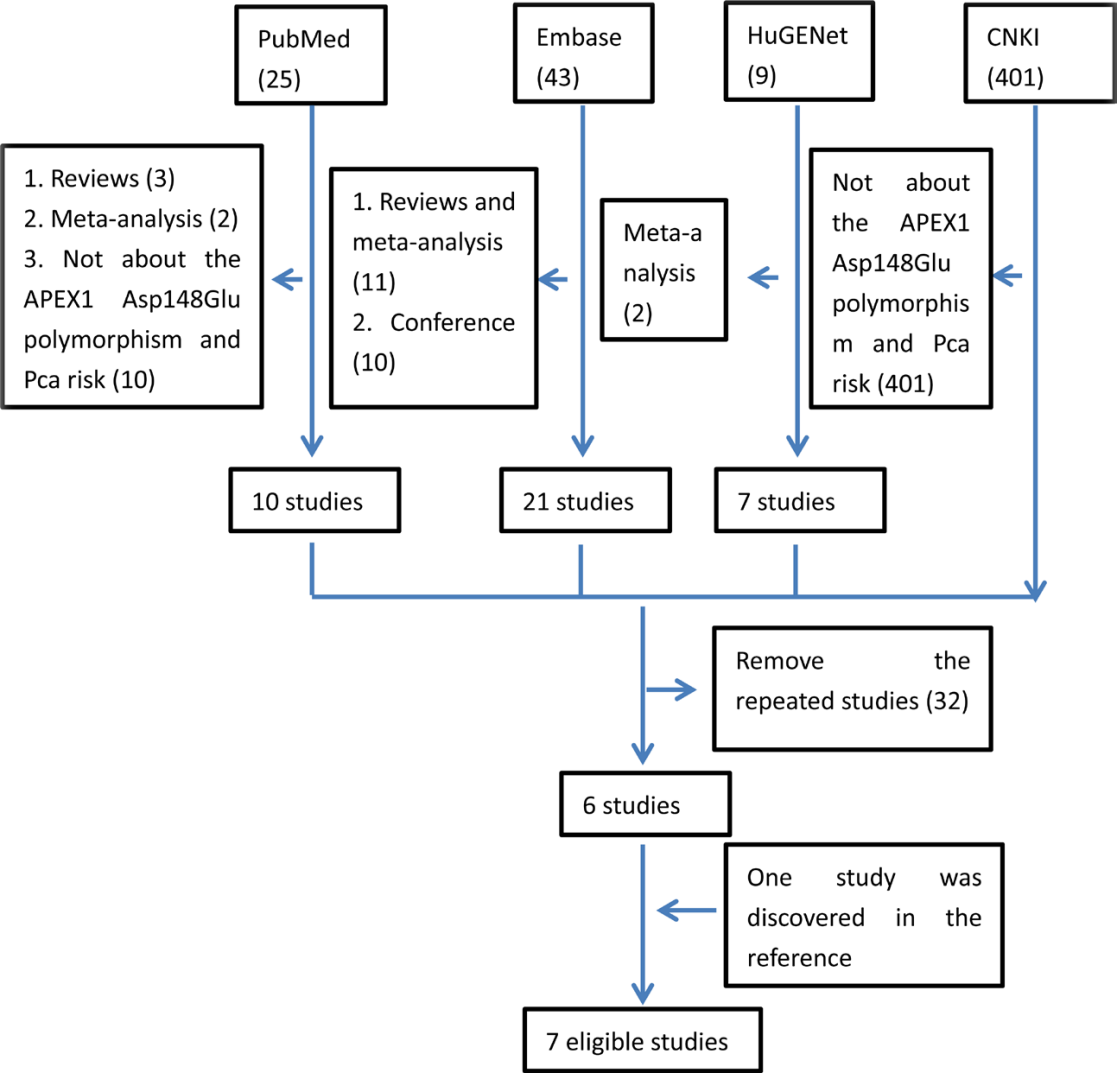
The flow of retrieval for this study

In the retrieval, four databases (PubMed, HuGENet, Embase and CNKI) were searched by combining the key words. Finally, 478 studies were included. When screening these studies by reading all the abstracts, 34 repeated studies in four databases were removed. Meanwhile, 437 studies were also excluded as there was no evidence of studying the association between Pca risk and APEX1 gene polymorphisms. Then, only seven studies were left. However, while scanning all the full texts, one study written by Agalliu et al. in 2010 was removed, as it was mainly focused on the association between the other APEX1 gene polymorphisms (rs1320150 and rs2275007) and Pca [19]. Moreover, by reading the references in the related studies carefully, one additional study was also found to be eligible [29]. Thus, seven articles with intact genotype data were included in the whole analysis [8, 17, 20–22, 29, 30]. The flow of retrieval was showed in Figure 1.

Among these eligible studies, most of the samples came from local medical institutions. Genomic DNA mainly came from the peripheral blood of the participants. Genotyping was conducted with polymerase chain reaction (PCR). Cases were defined as Pca patients who had been confirmed by histopathological examination. Cancer-free and healthy subjects were identified as the controls, who were matched to the cases by age or other characteristics. Among the eligible studies, three included subjects of Asian descentfrom China, Iran, and India [8, 20–21, 30]. Two from Brazil and America included subjects of mixed race, mostly European and African descent, and others involved [17, 22]. The last study included subjects of African descent(including self-identified African American, East African, West African, and Afro-Caribbean subjects) from America [29]. Among the eligible studies, samples in the Chen et al. [17] study could be divided into black (which could be classified into African descent) and white (which could be classified into mixed descent or Caucasian), in which the black subjects could be combined with the subjects of the Lavender et al. [29] study. So, in this analysis, three ethnic subgroups were defined as follows: Asian descent (Pournourali et al. [30], Jing et al. [8], Mandal et al. [20] and Mittal et al. [21]), African descent (Lavender et al. [29] and Chen et al. [17]) and mixed descent including European and African (Chen et al. [17] and Kuasne et al. [22]). After analysis, a significant association was discovered which had not been reported before.

**Table 1:** the essential characteristics of the eligible studies included in this analysis

Author	Years	Country	Race	Case	Control	Method	caseG	caseT	controlG	controlT	N.case	N.control
Pournourali M et al	2015	Iran	Asian	Pca	disease-free control subjects	PCR-RFLP	110	90	90	110	100	100
Jing B et al	2013	China	Asian	Pca confirmed by histopathological examination	without cancers matched to the case group by age, diet or life style	PCR-RFLP	166	230	119	193	198	156
Mandal RK et al	2012	India	Asian	histologic presence of adenocarcinoma of the prostate in the biopsy specimen	cancer free, unrelated, age matched healthy control individuals of similar ethnicity	ARMS-PCR	101	283	118	330	192	224
Mittal RD et al	2012	India	Asian	histologically confirmed Pca	cancer free, unrelated, age and sex matched healthy individuals of similar ethnicity	ARMS-PCR	102	288	127	373	195	250
Kuasne H et al	2011	Brazil	Mix	suspicious findings on a digital rectal examination (DRE) and/or elevated PSA serum levels (C2.5 ng/ml), followed by histopathological confirmation of prostate cancer	cancer-free men with negative DRE and serum levels of PSA levels lower than 2 ng/ml, matched to cancer patients on the basis of age (±5 years), ethnic group (Euro and African descendents), and drinking and smoking habits	PCR-RFLP	93	251	68	276	172	172
Lavender NA et al	2010	America	African	histological confirmation of Pca	healthy volunteers	TaqMan-PCR	120	252	445	817	186	631
Chen L et al	2006	America	Mix	primary adenocarcinoma of the prostate	without cancers	PCR-RFLP	302	400	261	397	351	329

* Pca: prostate cancer; PCR: Polymerase Chain Reaction; PCR-RFLP: polymerase chain reaction-restriction fragment length polymorphism; ARMS: amplification refractory mutation specific; N.case: the total number of cases; N.control: the total number of controls.

* Kuasne H et al. : Mix descent included European and African descents.

* Chen L et al. : Mix descent included 240 black (which could be classified into African descent) and 447 white (which could be classified into Mix descent or Caucasians)

* Lavender et al. : African descent included African-Americans, West Africans, East Africans and Caribbeans

### APEX1 Asp148Glu polymorphism associated with Pca

**Figure 2:**
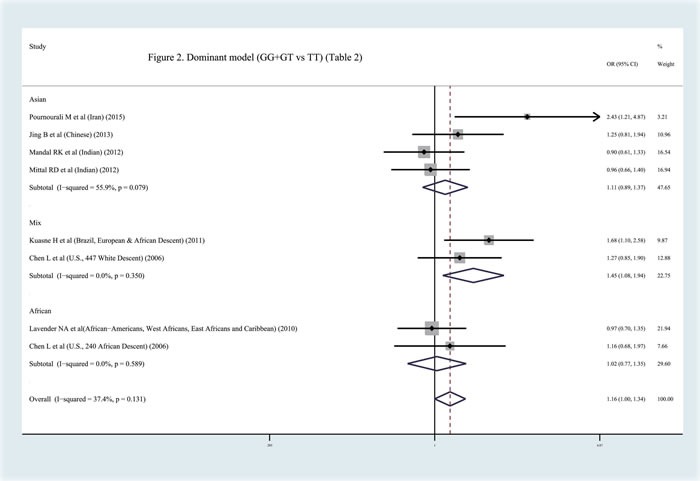
Meta-analysis with fixed effects and dominant model (GG+GT *vs* TT) for the association between APEX1 Asp148Glu polymorphism and the prostate cancer risk The first author and year of publication for each study was shown. In this analysis, three subgroups were shown: Asian descent, African descent and Mix races. OR and accompanying 95% CI were also presneted for this association.

**Figure 3:**
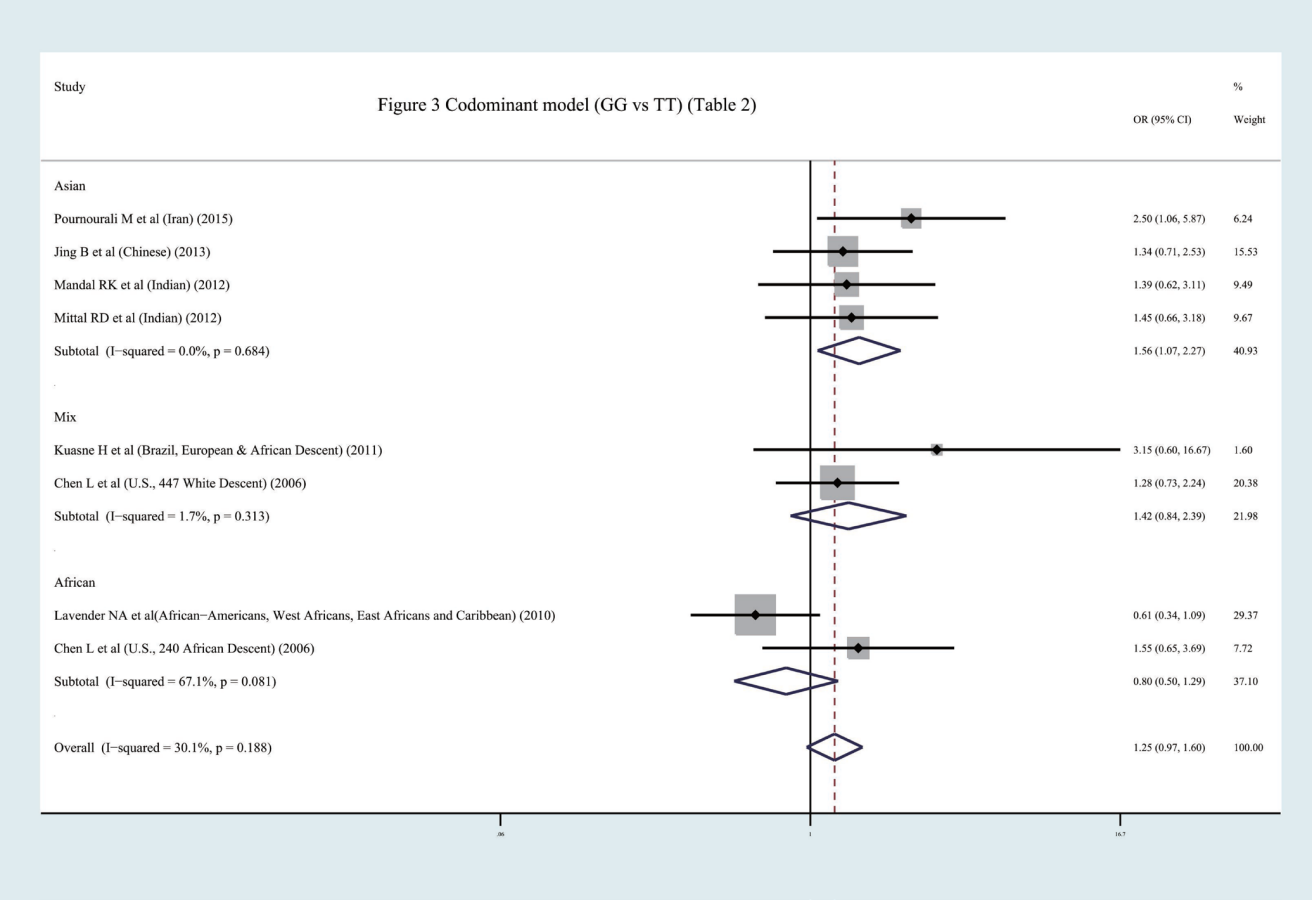
Meta-analysis with fixed effects and Codominant model (GG *vs* TT) for the association between APEX1 Asp148Glu polymorphism and the prostate cancer risk The first author and year of publication for each study was shown. In this analysis, three subgroups were shown: Asian descent, African Descent and Mix races. OR and accompanying 95% CI were also presneted for this association.

**Figure 4:**
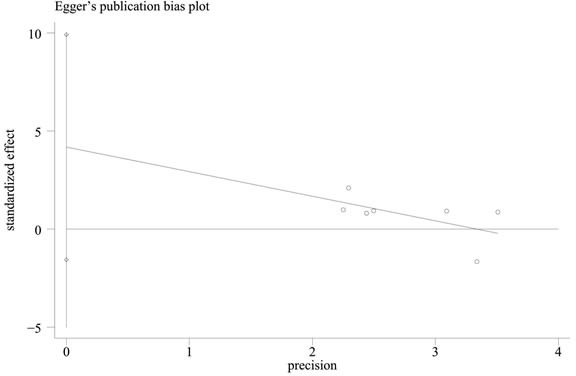
Funnel plot of publication bias for the co-dominant model (GG *vs* TT) in the meta-analysis with Egger's test

In contrast to previous meta-analyses [31–32], this study discovered a significant relationship between the APEX1 Asp148Glu polymorphism and Pca risk in the pooled results (dominant model: OR = 1.159, 95%CI = 1.000-1.344, *I^2^* = 37.40%, statistical power = 70.90%) (Figures 2–3, Table 2). In order to identify the possible association, a subgroup analysis was conducted. At this stage, the samples were divided into subjects of Asian descent, mixed descent and African descent. Although there were no positive results in the African group, our results suggested that the APEX1 Asp148Glu polymorphism was significantly related to Pca in the subjects of mixed descent (dominant model: OR = 1.450, 95%CI = 1.081-1.943, *I^2^* = 0.00%; per-allele analysis: OR = 1.261, 95%CI = 1.019-1.559, *I^2^* = 32.00%), which had been demonstrated previously [32](Figure 2, Table 2). Meanwhile, a potential association between the APEX1 Asp148Glu polymorphism and Pca risk among subjects of Asian descentwas also discovered, which had not been reported before (co-dominant model (GG *vs*. TT): OR = 1.557, 95%CI = 1.069-2.268, *I^2^* = 0%, statistical power = 65.90%) (Figure 3, Table 2). Next, the Hardy-Weinberg equilibrium (HWE) of each study was taken into consideration; one study did not satisfy HWE and was removed (*P* = 0.0232) [22]. After re-analysis, the positive association disappeared among subjects of mixed descent and all pooled ethnic backgrounds. A publication bias for the co-dominant model (GG *vs*. TT) is shown in Figure 4 (*P* = 0.120), which further confirmed that the association among subjects of Asian descent was reliable to some extent.

**Table 2:** Results after meta-analysis with dominant model (GG+GT vs TT), recessive model (GG vs GT+TT), codominant model (GG vs GT; GG vs TT) and per-allele analysis (G vs T) involved

Subgroups	Model	OR	95% CI	I2	P	Statistical Power	Authors
Asian Descent	Dominant model (GG+GT vs TT)	1.106	0.891, 1.374	55.90%	0.079	32.70%	Pournourali etal., Jing et al., Mandal et al., Mittal et al.
Asian Descent	Recessive model (GG vs GT+TT)	1.347	0.957, 1.895	0.00%	0.956	59.90%	
Asian Descent	Codominant model (GG vs GT)	1.266	0.885, 1.811	0.00%	0.736	7.60%	
Asian Descent	Codominant model (GG vs TT)	1.557	1.069, 2.268	0.00%	0.684	65.90%	
Asian Descent	Per-allele analysis (G vs T)	1.130	0.962, 1.326	0.00%	0.41	59.70%	
Mix Descent	Dominant model (GG+GT vs TT)	1.450	1.081, 1.943	0.00%	0.35	70.90%	Kuasne et al., Chen et al.
Mix Descent	Recessive model (GG vs GT+TT)	1.189	0.744, 1.900	0.00%	0.342	12.10%	
Mix Descent	Codominant model (GG vs GT)	1.073	0.656, 1.753	0.00%	0.468	5.00%	
Mix Descent	Codominant model (GG vs TT)	1.415	0.837, 2.393	1.70%	0.313	33.30%	
Mix Descent	Per-allele analysis (G vs T)	1.261	1.019, 1.559	32.00%	0.225	58.40%	
African Descent	Dominant model (GG+GT vs TT)	1.021	0.771, 1.352	0.00%	0.589	11.10%	Lavender et al., Chen et al.
African Descent	Recessive model (GG vs GT+TT)	0.771	0.495, 1.200	71.10%	0.063	21.90%	
African Descent	Codominant model (GG vs GT)	0.745	0.468, 1.183	69.40%	0.071	32.00%	
African Descent	Codominant model (GG vs TT)	0.803	0.501, 1.287	67.10%	0.081	11.00%	
African Descent	Per-allele analysis (G vs T)	0.954	0.778, 1.171	38.30%	0.203	5.00%	
All	Dominant model (GG+GT vs TT)	1.159	1.000, 1.344	37.40%	0.131	70.90%	Pournourali etal., Jing et al., Mandal et al., Mittal et al., Kuasne et al., Chen et al., Lavender et al
All	Recessive model (GG vs GT+TT)	1.112	0.882, 1.401	14.00%	0.32	16.30%	
All	Codominant model (GG vs GT)	1.043	0.818, 1.330	11.70%	0.339	5.30%	
All	Codominant model (GG vs TT)	1.246	0.968, 1.605	30.10%	0.188	99.80%	
All	Per-allele analysis (G vs T)	1.108	0.995, 1.235	25.90%	0.222	62.20%	

* Three subgroups were divided: Asian descent, Mix descent and African descent.

* OR= odd ratio; CI: confidence interval.

* The Statistical Power was calculated with the Power and Precision V4 software (http://www.power-analysis.com/).

### Sensitivity analysis

In order to confirm the association between the APEX1 Asp148Glu polymorphism and Pca risk after considering the HWE, a sensitivity analysis was conducted by excluding each study one at a time individually for every genotype model. To our surprise, a positive association emerged again in the co-dominant model (GG *vs*. TT) (Asian descent: OR = 1.557, 95%CI = 1.069-2.268, *I^2^* = 0.0%, statistical power = 65.90%; pooled descent: OR = 1.474, 95%CI = 1.099-1.976, *I^2^* = 0.0%; *P_for publication bias_* = 0.150, statistical power = 9.40%) and the per-allele analysis (G *vs*. T) (pooled descent: OR = 1.137, 95%CI = 1.000-1.294, *I^2^* = 0.0%; *P _for publication bias_* = 0.223, statistical power = 75.00%) after removing the same study [29].

### Gleason score, prostate-specific antigen and clinical status

**Figure 5:**
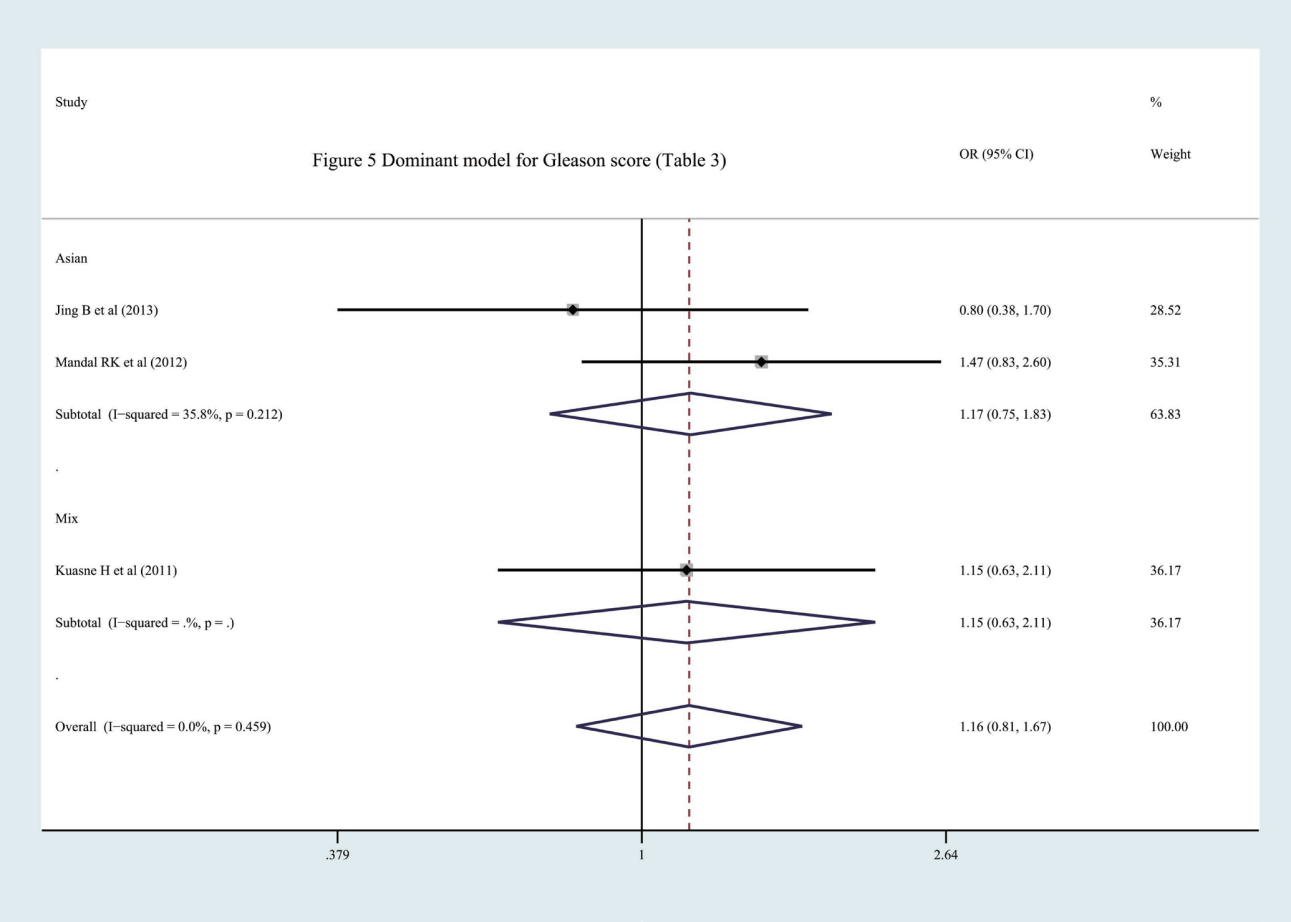
Meta-analysis with fixed effects and Dominant model (GG+GT *vs* TT) for the association between APEX1 Asp148Glu polymorphism and the prostate cancer risk(Gleason score {Greater than or equal to} 7) with three studies The first author and year of publication for each study was shown. In this analysis, three subgroups were shown: China, India and Mixed.OR and accompanying 95% CI were presneted for this association.

In this part of the study, we tried to investigate the function of the APEX1 Asp148Glu polymorphism in the development of Pca, on the basis of the Gleason score, prostate-specific antigen expression and clinical status of the cancer. Three studies with related information were included [8, 20, 22]. Two of them included subjects of Asian descent[8, 20] and one included subjects of mixed descent with data on only the dominant modl (GG+GT *vs*. TT) [22]. After analysis, we did not observe any significant relationship between the APEX1 Asp148Glu polymorphism and Pca invasiveness(Figure 5, Table 3).

**Table 3:** Association between APEX1 Asp148Glu polymorphism and three main indexes about the Pca development (GS, clinical status and PSA level)

Index	Race	Model	OR	95% CI	I2	P	
GS	Asian Descent	Dominant model (GG+GT vs TT)	1.169	0.745, 1.834	35.80%	0.212	Jing et al., Mandal et al.
GS	Asian Descent	Recessive model (GG vs GT+TT)	1.078	0.644, 1.807	31.70%	0.226	
GS	Asian Descent	Codominant model (GG vs GT)	0.898	0.265, 3.049	70.40%	0.066	
GS	Asian Descent	Codominant model (GG vs TT)	0.876	0.454, 1.691	0.00%	0.704	
GS	Asian Descent	Per-allele analysis (G vs T)	1.109	0.811, 1.517	0.00%	0.784	
GS	Mix Descents	*Dominant model (GG+GT vs TT)	1.164	0.811, 1.669	0.00%	0.459	Jing et al., Mandal et al., Kuasne et al.
clinical status	Asian Descent	Dominant model (GG+GT vs TT)	1.06	0.303, 3.712	87.40%	0.005	Jing et al., Mandal et al.
clinical status	Asian Descent	Recessive model (GG vs GT+TT)	1.53	0.911, 2.569	0.00%	0.593	
clinical status	Asian Descent	Codominant model (GG vs GT)	1.335	0.737, 2.418	0.00%	0.658	
clinical status	Asian Descent	Codominant model (GG vs TT)	1.747	0.955, 3.195	40.60%	0.194	
clinical status	Asian Descent	Per-allele analysis (G vs T)	1.109	0.469, 2.625	86.30%	0.007	
clinical status	Mix Descents	*Dominant model (GG+GT vs TT)	0.903	0.416, 1.963	78.30%	0.01	Jing et al., Mandal et al., Kuasne et al.
PSA	Mix Descents	*Dominant model (GG+GT vs TT)	0.897	0.560, 1.437	0.00%	0.62	Jing et al., Kuasne et al.

* The results of “*Dominant model (GG+GT vs TT)” contained three sets of data (reference 8, 20, 22) with mix races.

* GS: Gleason score; PSA: prostate-specific antigen

* GS groups: the high vs. low Gleason score (Gleason≥7 versus Gleason <7)

* Clinical status groups: case: Advanced or Metastasis (+) or clinical stages≥T3; control: Localized or Metastasis (−) or clinical stages<T3

* PSA group modeled as a categorical variable. The threshold was 10ng/ml (PSA case: PSA>10 ng/mL, PSA control: PSA≤10 ng/mL)

## DISCUSSION

Prostate cancer is a serious cancer that affects a large number of patients. As a multi-factor disease, genetic mutation has been found to play a key role in its development and progression. Recently, the APEX1 Asp148Glu (rs1130409) polymorphism was found to be associated with Pca risk [30]. However, this conclusion was controversial. In 2014, Li et al. investigated the association between rs1130409 and Pca [32]. Then, based on the same data, a new meta-analysis was conducted, which had discovered the other association in special ways [31]. Although the two meta-analyses focused on the association between APE1 polymorphisms and Pca in two aspects, our study was conducted in a different way to investigate the potential relationship more comprehensively. First, we updated the meta-analysis with studies from various databases (PubMed, Embase, HuGENet and CNKI); one additional study conducted by Pournourali et al. [30] was included. Secondly, in our meta-analysis, the ethnicities of the subjects were divided into three parts (Asian descent, mixed descent and African descent) in order to understand the association between APEX1 polymorphisms and Pca more in more detail in different populations. After the analysis, a potential relationship was found among the mixed descent subjects (a mix of European descent, African descent, and others) and subjects of Asian descent, which had not been reported in the previous studies [31–32]. In addition, we also conducted an additional valuable analysis, which was mainly focused on the function of the APE1 polymorphism in the development of Pca. In this part of the study, the Gleason score, prostate-specific antigen expression and clinical status of the cancer were included to evaluable the status of Pca. Although we did not obtain any positive results, this result suggests that rs1130409 might be a hereditary factor associated with Pca that promotes the development rather than the invasion of Pca to some extent.

APEX1 is an important gene encoding for DNA (apurinic or apyrimidinic site) lyase, which belongs to the BER pathway activating DNA repair, the cell cycle, and apoptosis [9–10, 12]. As the most frequently assessed variant, the APEX1 Asp148Glu polymorphism is thought to be associated with many cancers [14–16, 23–25, 27]. As for its biological function, APEX1 is related to a greater sensitivity to ionizing radiation, thus inducing mitotic delay in lymphocytes [33]. In order to describe the characteristics of this locus, we performed TagSNP, linkage disequilibrium (LD) and SNP function analysis with Haploview 4.2 and SNP Function Prediction (FuncPred) (http://manticore.niehs.nih.gov/snpinfo/snpfunc.htm), which suggested that the APEX1 Asp148Glu polymorphism is a tag- and functional SNP in the LD block with other SNPs in the APEX1 gene. In addition, as a missense mutation, we have reason to believe that the APEX1 Asp148Glu polymorphism is an important locus to study.

In 2013, Mahjabeen et al. [23] showed that APEX1 mutations and the deregulation of APEX1 are associated with an increased risk of head and neck cancer in the Pakistani population. Meanwhile, Li et al. [24] identified that polymorphisms of the APEX1 gene might contribute to tumorigenesis in lung cancer among the Chinese population. In addition, the APEX1 gene could also increase the risk of glioblastoma [25], gastric cancer [26], and bladder cancer [27]. Recently, more studies have shown that the APEX1 gene plays a key role in the development and progression of Pca. In 2001, Kelley et al. [28] studied the function of the Ape1/ref-1 enzyme in the risk of Pca, which suggested that this enzyme might be a diagnostic marker for early Pca and play a role in the physiology of the early development of this disease. As one of the important polymorphisms of APEX1 gene, rs1130409 has been suspected to be significantly associated with Pca. In 2006, Chen et al. [17] first studied the relationship between the APEX1 Asp148Glu polymorphism and Pca in white and black Americans, and identified the positive function of this polymorphism in determining the risk of Pca. Then, in 2011, Kuasne et al. [22] confirmed this significant association again in Brazilian men. However, there were no publically available GWAS databases, and GWAS analysis had evaluated this SNP and Pca outcomes before. Recently, two meta-analyses were conducted that showed a significant association between the APEX1 Asp148Glu polymorphism and Pca, especially among Caucasian subjects and the hospital-based population [31–32]. In order to discover the real association, this analysis was conducted. Although a positive association between the APEX1 Asp148Glu polymorphism and Pca was presented in the pooled and special subgroups analysis (mixed and Asian descent), after considering the HWE, the significant association was disappeared among all pooled and mixed descents. In order to confirm these associations, a sensitivity analysis was performed by removing the studies one by one, which suggested that the APEX1 Asp148Glu polymorphism should be associated with Pca, especially in subjects of Asian descent. In addition, we directed our attention to the invasion of Pca. After a comprehensive analysis, we could not identify an explicit relationship. So, on the basis of this analysis, we suggest that the APEX1 Asp148Glu polymorphism might be more important in the development of Pca rather than invasion. In order to confirm these results, the Gene Expression Omnibus (GEO) (https://www.ncbi.nlm.nih.gov/geo/) was applied, in which GSE76470 was used. GSE76470 is mainly focused on the differences in gene expression between epithelial-like (EL) clones with high tumorigenic ability. In other words, EL clones appear to be more aggressive compared to mesenchymal-like (ML) Pca cells. After analysis, we found that there was no statistical difference between aggressive and non-aggressive Pca regarding APEX1 gene expression (adjusted *P*-value = 0.87091, logFC = 0.07395), which might explain the reason for the lack of an association between the APEX1 Asp148Glu polymorphism and invasive Pca to some extent.

Combining the features and function of the APEX1 Asp148Glu polymorphism, recent studies and our comprehensive analysis, we suggest that the rs1130409 mutation might increase the risk of developing Pca, especially among Asian subjects.

## LIMITATIONS

After a comprehensive meta-analysis, a relationship between Pca and the APEX1 Asp148Glu polymorphism was shown. However, there are still some limitations. Firstly, the number of studies on the association between the APEX1 Asp148Glu polymorphism and Pca is limited. We cannot rule out a significant relationship between SNP and Pca outcomes, which may be attributed to the lack of statistical power to observe significant relationships to some extent. Secondly, the populations in the studies were different, especially regarding the mixed race subgroup, which could result in some heterogeneity in the results. Thirdly, there were three polymerase chain reaction (PCR) methods applied, namely PCR-restriction fragment length polymorphism (PCR-RFLP) analysis, amplification refractory mutation specific PCR (ARMS-PCR) analysis and TaqMan-PCR. The genotype misclassification for each method might affect the risk estimates in some way. Finally, research into the mechanism of the APEX1 Asp148Glu polymorphism in the development and progression of Pca was limited, especially regarding interactions with other BER pathway genes and the environment.

## CONCLUSIONS

Prostate cancer is a cancer of global concern. The APEX1 Asp148Glu polymorphism is thought to be associated with Pca risk; this study was conducted in order to make this relationship clearer. Our results suggest that the APEX1 Asp148Glu polymorphism is significantly associated with Pca in subjects of Asian descent as well as in other populations, and might stimulate the development of Pca rather than invasion. Additional larger and ethnically diverse studies are needed to confirm the real relationships and functions.

## MATERIALS AND METHODS

### Searching and collecting

Our eligible studies were mainly retrieved from the PubMed, Embase, HuGENet and Chinese National Knowledge Infrastructure (CNKI) databases. In order to collect more comprehensive eligible studies, we conducted an extensive retrieval, with the following keywords combined: “apurinic/apyrimidinic endonuclease”,“APE1”, “APEX1”, “APEN”, “HAP1”, “Pca”, “prostate cancer”, “prostatic carcinoma”, and “prostate carcinoma”. All studies identified were original research articles and were written in English or Chinese. The last search was performed in October 2015. In order to select the eligible studies, we set the following inclusion criteria: (1) the essential contents were about the APEX1 Asp148Glu polymorphism and Pca; (2) studies were designed in case-control or cohort format, which could distinguish the case group and controls; (3) the number of cases and controls for separate genotypes should be provided. In other words, sufficient information for estimating the OR and the 95% CI should be provided.

After selecting the included studies, we summarized the population characteristics of all seven studies for our meta-analysis, including the author's name, publication year, country, ethnicity (categorized as Asian descent, African descent or mixed descent), definition of cases and controls, allelic discrimination method, and numbers of cases and controls, as shown in Table 1.

### Statistical analysis

Frequencies of every genotype were included in the next analysis. In order to describe the association between the APEX1 Asp148Glu polymorphism and Pca risk, we selected four common genotype models, including the dominant (GG + GT *versus* TT), recessive (GG *versus* GT + TT), co-dominant model (GG *versus* GT; GG *versus* TT) and per-allele analysis (A *versus* C). As for the effect model, *I^2^* was treated as standard (when *I^2^* < 50%, the fixed effect was applied; otherwise, the random effect would be selected). The effects incorporated an estimate of the inter-study variance and provided wider 95% confidence intervals (95% CI) if the results of the constituent studies differed among themselves. In all the analyses, the relationship between the APEX1 Asp148Glu SNP and Pca were estimated using odds ratios and the corresponding 95%CI. The current study estimated both population-specific and pooled risk estimates. Meanwhile, the chi-squared-based Q statistic (*P* < 0.10 as the standard) was applied to estimate heterogeneity, which represented the weighted sum of the squared difference in the overall effect sizes from each study [18]. Then, we conducted the subgroup analysis by collecting similar characteristics form the eligible studies, such as country of origin and ethnicity. In this meta-analysis, five countries were represented: China, India, Iran, Brazil and America. As for the races, three groups were represented: Asian descent, African descent and mixed descents. Then, the Hardy-Weinberg equilibrium (HWE) for each study was taken into consideration. This process of analysis was conducted after removing studies that did not satisfy HWE to confirm our results.

Meanwhile, in order to understand the function of the APEX1 Asp148Glu polymorphism in Pca development, a further analysis was conducted. In this part of the study, three indexes [Gleason score (GS), clinical status and the level of prostate-specific antigen (PSA)] were considered. After re-reading the included studies, three of them were found to contain the necessary data and were analyzed [8, 20, 22]. For GS and PSA, two groups were generated based on the threshold of 7 (GS case: GS≥7, GS control: GS < 7) and 10 (PSA case: PSA > 10 ng/mL, PSA control: PSA≤10 ng/mL), respectively. In addition, according to the invasiveness of the cancer, another two groups were also defined (case: advanced or metastasis (+) or clinical stage≥T3; control: localized or metastasis (−) or clinical stage < T3).

Publication bias was evaluated with Egger's linear regression test and a funnel plot. The statistical power was calculated with Power and Precision V4 software (http://www.power-analysis.com/). All *P* values were two-tailed, and data were analyzed using Stata 9.0 (Stata Corporation, USA).
